# First-line medical thoracoscopy for pleural infection: the SPIRIT randomised controlled feasibility trial

**DOI:** 10.1136/bmjresp-2025-003675

**Published:** 2025-12-10

**Authors:** Krishan Ragab Bansal, David T Arnold, Emma Tucker, Anna Morley, Liju Ahmed, Hugh Ip, Parthipan Sivakumar, Henry Steer, Matthew Evison, Najib Rahman, Mohammed Munavvar, Kevin G Blyth, Justin Pepperell, Nick Maskell, Rahul Bhatnagar

**Affiliations:** 1 Respiratory Medicine, University of Bristol Academic Respiratory Unit, Bristol, UK; 2 Southmead Hospital Respiratory Medicine, Bristol, UK; 3 Respiratory Medicine, Southmead Hospital Respiratory Medicine, Bristol, UK; 4 Guy's and St Thomas’ Hospitals NHS Trust, London, UK; 5 King Faisal Specialist Hospital and Research Centre, Riyadh, Saudi Arabia; 6 Royal Free Hospital, London, UK; 7 Blacktown Hospital, Blacktown, New South Wales, Australia; 8 Respiratory Medicine, Gloucestershire Hospitals NHS Foundation Trust, Gloucester, UK; 9 Wythenshawe Hospital, Manchester, UK; 10 Manchester Academic Health Science Centre, Manchester, UK; 11 Oxford Respiratory Trials Unit, Oxford, UK; 12 NIHR Oxford Biomedical Research Centre, Oxford, UK; 13 Royal Preston Hospital, Preston, UK; 14 University of Central Lancashire School of Medicine, Preston, UK; 15 University of Glasgow Institute of Cancer Sciences, Glasgow, UK; 16 Queen Elizabeth University Hospital, Glasgow, UK; 17 Musgrove Park Hospital, Taunton, UK

**Keywords:** Pleural Disease, Respiratory Infection

## Abstract

**Background:**

Pleural infection remains a significant clinical challenge, requiring hospitalisation, intravenous antibiotics and early chest drain insertion. Medical thoracoscopy (MT), a minimally invasive procedure used electively in the UK for malignant effusions, has demonstrated good outcomes when applied to acute pleural infection in retrospective case series. However, it has not been evaluated as a first-line intervention in the UK in a randomised controlled trial (RCT).

**Objectives:**

The Studying Pleuroscopy in Routine Pleural Infection Treatment (SPIRIT) trial assessed the feasibility of conducting a full-scale RCT comparing MT with chest drain insertion for acute pleural infection within UK National Health Service (NHS) hospitals.

**Methods:**

SPIRIT was an open-label, randomised feasibility trial conducted across seven NHS centres between 2017 and 2019. Adults with suspected pleural infection were prescreened; eligible patients were randomised to either chest drain insertion (control) or MT (performed the same or following day) with 90-day follow-up. The primary outcome was feasibility, assessed through a composite of prescreen, screen and allocation failure rates. Secondary outcomes included inpatient-stay duration, mortality, radiological and microbiological outcomes, second-line interventions, patient-reported outcomes and adverse events.

**Results:**

Of 193 patients prescreened, 181 (93.8%) were excluded due to at least one criterion. Key factors included lack of MT deliverability (49.2%), a not truly infected effusion (45.1%) and contraindications to drainage or study involvement (44.0%). Consequently, the primary feasibility endpoint was not met. All 12 eligible patients were randomised with no attrition. MT lasted 15 min longer than drain insertion, but chest drains remained in situ over 3 days longer (p=0.17) with a longer hospital stay (p=0.57). Radiological improvement, microbiological yield and symptom scores were similar. Adverse events occurred in one control and three MT patients.

**Conclusion:**

A full-scale RCT is not likely to be feasible in an NHS setting on the proposed protocol. Targeted recruitment from centres equipped for emergency MT may enhance feasibility.

**Trial registration number:**

ISRCTN98460319.

**WHAT IS ALREADY KNOWN ON THIS TOPIC**

- Pleural infection requires urgent chest drain insertion; however, some studies report success with early medical thoracoscopy (MT) for pleural drainage. UK MT centres provide elective services; hence, it is unclear whether they are suitable for first-line drainage in pleural infection.

**WHAT THIS STUDY ADDS**

- Studying Pleuroscopy in Routine Pleural Infection Treatment demonstrates that safely delivering a randomised controlled trial using the existing protocol approach is currently unfeasible in a UK National Health Service (NHS) setting.
- Key reasons included a lack of provision for urgent MT, patients either not having capacity to provide informed consent or being unwilling to receive information about the study and challenges delivering postprocedure care.

**HOW THIS STUDY MIGHT AFFECT RESEARCH, PRACTICE OR POLICY**

- This study may inform the design of future randomised trials in pleural infection or relating to first line MT.
- Reconsideration of recruitment criteria may allow for a more pragmatic evaluation of MT in an NHS setting when managing pleural infection.

## Introduction

 Pleural infection, including empyema, affects approximately 15 000 individuals annually in the UK^1^ and is associated with considerable morbidity and a mortality rate of 10–20%, particularly in frail or immunosuppressed patients.^2^,^5^ Median hospital stay is 13 days,^6^ with most patients requiring urgent chest drain insertion and prolonged intravenous antibiotics. Extended admissions typically reflect failure of standard management, often due to loculated pleural fluid with fibrin deposition. Second-line therapies such as intrapleural enzyme therapy (IET) or video-assisted thoracoscopic surgery (VATS) are then considered; first-line surgery is rare.

MT, also known as local anaesthetic thoracoscopy or pleuroscopy, is widely used in the UK.^7^ Performed under moderate sedation, MT allows for maximal fluid drainage, septation breakdown, pleural biopsy and chest drain placement.^8^ The procedure has a low major complication rate (1.8%) and negligible mortality (0.3%).^9^ Patients often require minimal inpatient recovery, making MT theoretically appealing for acute pleural infection management.

Several European and US studies in select centres have reported successful use of MT in this context.^10^,^13^ However, there are no comparative studies to inform the utility of MT as an early intervention for pleural infection. Despite widespread availability, MT in the UK is primarily reserved for managing malignant pleural effusion (MPE), typically on elective or semielective scheduled lists. It often occurs in set locations such as theatre or endoscopy suites, and its use in an emergency care pathway remains limited.

The Studying Pleuroscopy in Routine Pleural Infection Treatment (SPIRIT) trial was designed to inform the design and explore the feasibility of a large scale and fully powered RCT of MT in acute early pleural infection, specifically in a UK National Health Service (NHS) setting.

## Methods

### Study design and setting

SPIRIT was an open label, randomised, feasibility study conducted at seven NHS hospitals between 2017 and 2019 with established pleural services and expertise in the delivery of MT. Centres were selected to provide both geographical variety and a range of procedure environments, including theatre suites, endoscopy units and ward pleural procedural rooms. A trial steering committee, with independent leadership and lay representation, approved study design and oversaw study conduct.

### Funding, approvals and registration

The study was funded by the Academy of Medical Sciences and received approval from the Yorkshire and the Humber NHS research ethics committee (17/YH/0074). The Sponsor was North Bristol NHS Trust. The study was prospectively registered at ISRCTN98460319 and was conducted in accordance with the principles of the Declaration of Helsinki.

### Participant identification

Participants were identified from routine and admission avoidance pleural clinics and inpatient reviews.

Patient prescreening identified those with suspected pleural infection, who were then assessed against the inclusion and exclusion criteria. Adults (≥18 years) with suspected pleural infection were eligible if they met at least one of the following: fluid pH ≤7.20 or visible pus; fluid glucose ≤3.4 mmol/L; positive bacterial or mycobacterial culture or positive gram or acid-fast bacilli stain.^2 3 14^ In addition, patients were only eligible if MT could be delivered on the same or following day to treatment allocation, minimising treatment delays for potentially unwell patients and mimicking usual chest tube insertion practice.

Patients were ineligible if they had any contraindication to MT under moderate sedation, chest drain insertion or trial involvement. They were also ineligible if they were not expected to survive for at least 3 months; were pregnant; did not have capacity to provide informed consent or were unwilling to receive information about this study; had ultrasound appearances suggesting a fluid depth of ≤2 cm or appearances not amenable to MT; had ongoing sepsis requiring support other than intravenous fluids or previous ipsilateral thoracic surgery within 6 months (or a previous lobectomy/pneumonectomy at any time). Centres were asked to document all reasons for ineligibility; hence, multiple reasons for exclusion were possible for each patient.

Those meeting the entry criteria were offered a patient information sheet and entered onto screening logs, with written informed consent obtained from those willing to enrol.

Patients with suspected pleural infection not meeting entry criteria were defined as ‘prescreen failures.’ Patients meeting all criteria but declining study entry were defined as ‘screen failures.’

See online supplemental file 1 for the study recruitment flowchart.

### Treatment allocation

Following consent, participants were randomised 1:1 via a web-based system, to either the control or intervention arm using open-label minimisation with a random component. The minimisation factors were fluid complexity on ultrasound (none/mild vs moderate/severe) and visually estimated size of effusion on chest radiograph (<50% vs ≥50% opacification). Procedures were to be performed on the same or following day to allocation, with separate procedure-specific consent obtained. Participants were classed as ‘allocation failures’ if the assigned procedure was not performed within this timeframe or at all.

### Trial procedures

#### Control (usual care) arm

Participants underwent ultrasound-guided chest drain insertion (≥16F gauge), using either dissection or a drain-over-wire (Seldinger) method. Drains were secured using at least one suture and connected to any standard drainage system. Fluid drainage rate was at the treating physician’s discretion. A chest radiograph was performed 12 hours post insertion.

#### Intervention arm

Participants underwent MT as per local hospital policy. Pneumothorax induction was permitted for small effusions. MT could be rigid or a semirigid technique, using single-port or dual-port access. The use of conscious sedation was at physician discretion.

When safe, operators were instructed to undertake breakdown of visible and accessible fibrinous septations; obtain parietal pleural biopsies and to perform an intrapleural washout using 0.9% saline.

Post procedure, as per the control arm, a chest drain (≥16F gauge) was inserted, secured and connected to a drainage system, with a chest radiograph at 12 hours.

Participants unable to undergo their allocation MT received standard chest drain insertion.

#### Postprocedure clinical management

Post procedure, clinical care followed local standard practice at the discretion of the treating physician, including the timing of chest drain removal and hospital discharge. Regular blood tests, thoracic ultrasounds and assessments of symptoms and quality of life were conducted during admission.

The use of second-line therapy (IET or referral for VATS) was permitted at the treating physician’s discretion. A guide for when to consider IET was provided to all sites for standardisation.

### Trial assessment schedule

Baseline assessments were completed on enrolment. Blood tests were performed at least on alternate days until day 9 post procedure and then weekly if still an inpatient. Visual Analogue Scale (VAS) scores and thoracic ultrasounds were collected on days 1, 3 and 7, then weekly as needed. The EuroQol-5 Dimensions-5 Level Version (EQ-5D-5L) quality-of-life questionnaire was completed on day 7. Chest radiographs were obtained at baseline, post procedure and discharge.

Participants were reviewed on days 30 and 90 post procedure, including clinical assessment, blood tests, chest radiograph, thoracic ultrasound and EQ-5D-5L completion. Radiographs were assessed independently by two blinded clinicians. Pleural effusion size and loculation were graded on ultrasound and by change in hemithorax opacification on a chest radiograph, a recognised and previously published method.^3^

### Outcomes

The primary outcome was feasibility, assessed by the ability to recruit, randomise and deliver trial procedures.

Secondary outcomes were inpatient stay duration, need for second line therapy (IET and/or thoracic surgery) and all-cause mortality. Adverse event types and numbers were captured along with chest radiograph changes and microbiological yield in both arms. Patient experience was measured using VAS for chest pain and breathlessness.

### Recruitment target and statistical analysis

As a feasibility study, no formal power or sample size calculation was required. However, a target of 30 patients was deemed sufficient to assess practicability and signals towards between-group differences.

The primary outcome (feasibility) was assessed as a three-part composite: <2/3 prescreen failure rate, <2/3 screen failure rate and <50% allocation failure rate. Overall study feasibility required success in each part.

Secondary outcomes were analysed on both an intention-to-treat and per protocol basis where possible. Categorical data were analysed using the χ^2^ approach and ordinal data using either the Mann-Whitney U test or an independent sample t-test.

### Patient and public involvement

Patient experiences were used as part of the secondary outcome analysis. Patients or the public were not involved in the design, conduct, reporting or results dissemination of this research.

## Results

### Patient demographics

**Figure 1:**
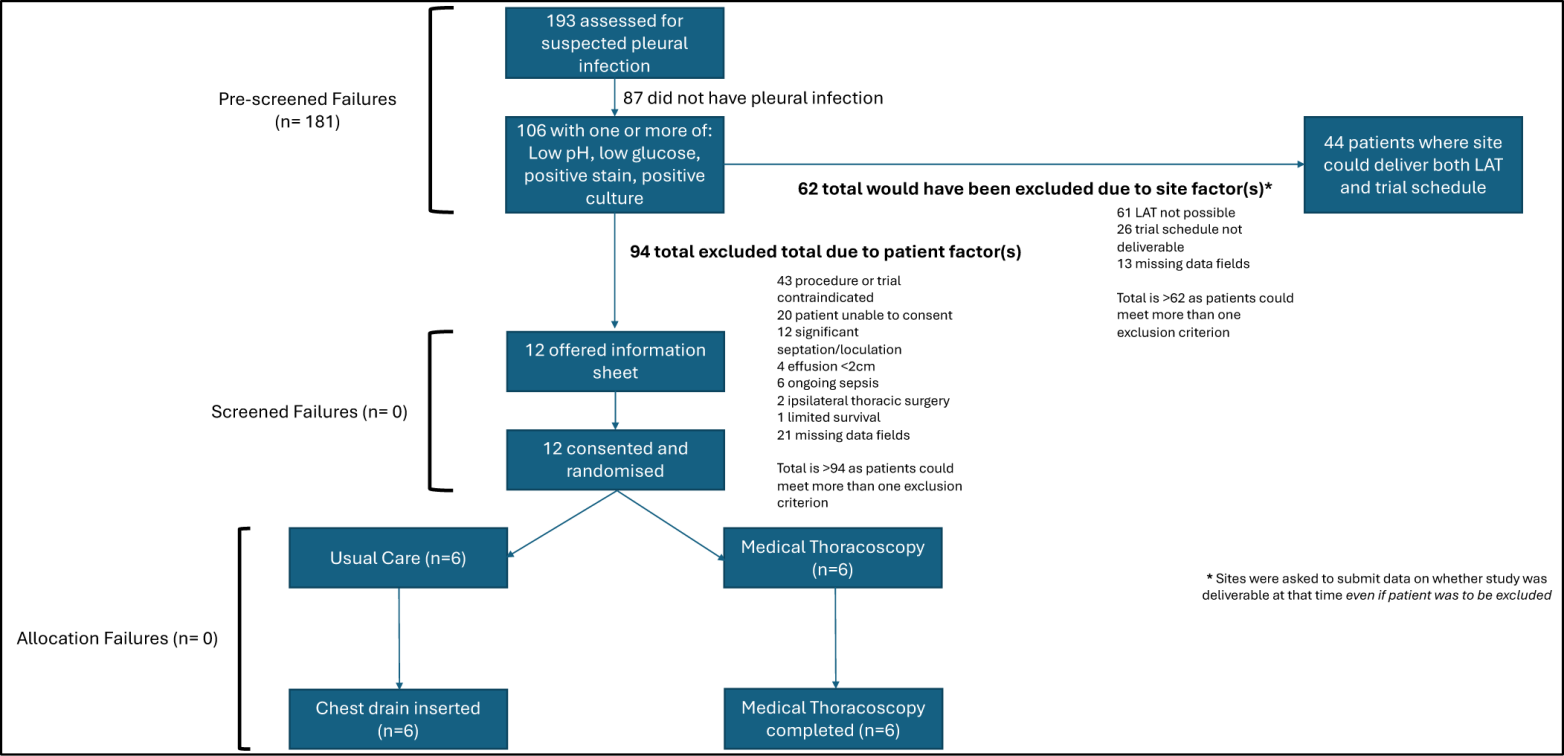
Consolidated Standards of Reporting Trials (CONSORT) diagram. LAT, local anaesthetic thoracoscopy (MT).

Between October 2017 and January 2019, 193 patients with a suspected infected pleural effusion were prescreened. After applying the inclusion and exclusion criteria, 12 of the target 30 participants proceeded to randomisation. All 12 received their allocated procedure (six in the control arm and six in the intervention arm) (see figure 1).

Six of the 12 participants (50%) were recruited from a single centre (site B) which had access to its own procedural suite. The remaining six centres who prescreened patients did not have access to a procedural suite and were instead reliant on endoscopy rooms or theatres. The location of centres of patient recruitment is described in table 1. The mean age of the 12 participants was 51 years (SD 15). Both groups were broadly similar at baseline, except for 10/12 (83.3%) being male. See table 2 for baseline demographic data and online supplemental file 2 for the complete data.

**Table 1:** Location of centres for participant recruitment

Site	Thoracoscopy location	Number of participants prescreened (n)	Number of participants in the control arm (n)	Number of participants in the intervention arm (n)
A	Theatre	27	1	0
B	Pleural suite	69	2	4
C	Theatre	9	0	0
D	Endoscopy	14	0	0
E	Endoscopy	20	1	1
F	Pleural suite	41	1	0
G	Endoscopy	13	1	1

**Table 2:** Demographics of trial entrants

	Control arm (n=6)	Intervention arm (n=6)	All (n=12)
Age	56 (13)	46 (16)	51 (15)
Male sex	5	5	10
Usual WHO performance status			
0	4	6	10
1	2	0	2
2	0	0	0
3	0	0	0
Current smoker	3	3	6
Ex-smoker	3	1	4
Alcohol excess	1	1	2
Lung disease	2	0	2
Heart disease	3	0	3
Diabetes mellitus	4	0	4
Laterality (R/L)	4/2	1/5	5/7
Mean fluid pH	6.93 (0.26)	7.12 (0.21)	7.02
Mean fluid protein	41.0 (4.3)	48.6 (22.2)	45.7
Mean fluid LDH	12 090 (18241)	5063 (8336)	7698
Mean fluid glucose	3.6 (5.8)	2.3 (1.7)	2.87
C reactive protein	208 (139)	213 (88.3)	211 (109)

Continuous data reported as means (SD).

LDH, Lactate dehydrogenase.

### Primary outcomes

Of the 193 patients with a suspected infective pleural effusion who were prescreened, 12 (6.2%) satisfied the trials’ inclusion and exclusion criteria, with the remaining 181 patients being deemed as prescreen failures. Patients often met multiple criteria for exclusion. 87 patients did not have a true infected pleural effusion as per the trial definition. In 95 patients, the centre was unable to deliver the intervention arm on the same day or next, and in 50 patients, the centre was unable to deliver the proposed postprocedural care schedule. 34 patients did not have capacity to provide informed consent or were unwilling to receive information about this study. Of the 85 with relative contraindications to either procedure or trial involvement, reasons were cited for 28. The most common were that the patient was too systemically unwell (including the presence of a pericardial effusion) (n=5), the infection related to an indwelling pleural catheter (n=3), the patient was too frail and/or they were managed palliatively (n=3) or there were concerns with their coagulation (n=2). The complete breakdown of prescreen failures is documented in table 3.

**Table 3:** Reasons for prescreen failures in this cohort

	Number of participants excluded for each criterion (n) (%)
Inclusion criteria	
True infected pleural effusion	87 (45.1%)
Ability for MT to be performed on the same day or next	95 (49.2%)
Ability to deliver postprocedural care as per the trial schedule	50 (25.9%)
Exclusion criteria	
Contraindication to (a) MT (b) chest drain insertion (c) patient trial involvement	85 (44.0%)
Patient unable to provide informed consent or unwilling to receive study information	34 (17.6%)
Fluid septation, loculation or an effusion position incompatible with MT or chest drain insertion	21 (10.9%)
Pleural fluid depth ≤2 cm on ultrasound	16 (8.3%)
Ongoing sepsis	5 (2.6%)
Ipsilateral thoracic surgery within 6 months	3 (1.6%)
Age <18 years	2 (1.0%)
Pregnancy or lactation	0 (0%)
Expected survival ≤3 months	6 (3.1%)
Total patients prescreened (n)	193
Total patients excluded as prescreen failures for meeting one or more of the above criteria (n)	181 (93.8%)

MT, medical thoracoscopy.

12 patients received study information, with 12/12 (100%) agreeing to consent (no screen failures). All 12 participants proceeded to randomisation; six patients were allocated to the control arm and six were allocated to the intervention arm. All 12 patients underwent their allocated procedures (no allocation failures).

Since the first of the three-part composite was unsuccessful, the primary outcome of feasibility was not met.

### Secondary outcomes

#### Procedure details and patient outcomes

Table 4 reports the variation in procedures and outcomes between the groups. Overall, the mean time taken for MT was 15 min longer (p=0.22). Mean (±SD) total drain output post procedure was nearly double in the control arm (n=1680±261 mL) compared with the intervention arm (n=763 ±873 mL) (p=0.69). Control arm participants had chest drains in situ for over 3 days longer (median=6.5, IQR=5.25–7 days) compared with the intervention arm (median=3, IQR=2.25–6 days) (p=0.17). Intervention arm participants had a longer length of stay in hospital (median=10 days, IQR=4–14 days) than the control arm (median=8 days, IQR=7–14 days) (p=0.57). One of the 12 participants (in the intervention arm) required thoracic surgery for control of pleural infection. Three participants (two in the control arm and one in the intervention arm) were treated with IET.

**Table 4:** Procedure, admission and treatment details

	Control arm (n=6)	Intervention arm (n=6)	All (n=12)	Between group difference (p values)
Procedure duration (min)	28 (12)	43 (25)	35 (20)	0.22
Volume removed during procedure (mL)	415 (370)	620 (544)	517 (455)	0.46
Total drain output (mL)	1680 (261)	763 (873)	1155 (802)	0.69
Days drain in situ, median (IQR)	6.5 (5.25–7)	3 (2.25–6)	5.3 (2.3)	0.17
Hospital length of stay (days), median (IQR)	8 (7–14)	10 (4–14)	8 (6–14)	0.57
Use of intrapleural fibrinolytics	2	1	3	0.51
Referred to thoracic surgery	0	1	1	0.30
Thoracic surgery performed	0	1	1	0.30
Duration of intravenous antibiotics days, median (IQR)	9 (2–15)	7 (5–11)	8 (5–11)	0.76
Patients requiring further oral antibiotics between discharge and day 30	3	3	6	1.00

Reported as mean (SD) unless otherwise specified.

At 30 days post procedure, one of 12 participants had died. This occurred in the control arm and their death (on day 23) was unrelated to the study intervention, attributed to an underlying lung adenocarcinoma which was not apparent at the time of enrolment. No further participants died during the 90 day follow-up. One participant in the control arm withdrew from the study on day 14. Two participants in the intervention arm were lost to follow-up at 90 days. The remaining eight patients completed follow-up for the full 90-day period.

#### Radiology

At baseline, five effusions were large (>50% of hemithorax), five moderate (25–50%) and two were small (<25%). Following allocated treatment, all participants showed a reduced pleural effusion size; nine of the 11 effusions had decreased to small or absent on discharge, and the remaining two effusions decreased to absent by day 30.

On thoracic ultrasound, eight of 12 effusions were mild or moderately loculated at baseline. By day 30 of follow-up, six of eight participants (with data available) demonstrated an improvement in their effusion size on ultrasound. By day 30 of follow-up, two participants in the intervention arm had mild persistent complexity.

For details regarding adherence to the imaging follow-up schedule, see online supplemental file 3.

#### Microbiology

Eight of the 12 participants (66.7%) (three in the control arm and five in the intervention arm) had a pleural fluid sample sent for microbiology, with one being positive for *Staphylococcus capitis* and a Gram-negative unidentified organism.

Of the six participants in the intervention arm, three (50.0%) had pleural tissue samples sent, one of which was positive for *Klebsiella pneumoniae*.

#### Health-related quality of life and patient-reported symptoms

Health-related quality of life improved following both procedures from baseline to follow-up at 90 days. Chest pain and breathlessness also improved from baseline following both procedures to follow-up at 30 days.

See online supplemental file 4 for the full results.

#### Blood results

The relevant blood results were broadly similar between the two study arms at baseline. 10 of the 12 participants (five in the control arm and five in the intervention arm) had blood results available at baseline, with a mean (±SD) C reactive protein (CRP) at baseline of 211.1±110.0 mg/dL. By day 30 and 90, all patients had a CRP of less than 20 mg/dL, with a mean (±SD) of 11.1 ±5.7 mg/dL.

#### Adverse events

Of the 12 participants, one severe adverse event was recorded (in the control arm). This referred to the patient who died on day 23 of a lung adenocarcinoma. No further adverse events were recorded in the control arm. 11 adverse events were recorded in the intervention arm, though eight occurred in one patient. Three patients in total from the intervention arm experienced adverse events, none of which were classified as severe. Online supplemental file 5 reports the complete data on adverse events reported in this trial.

## Discussion

SPIRIT, a multicentre feasibility study, compared MT with chest drain insertion for initial pleural infection management. Despite prescreening almost 200 patients across experienced centres, recruitment proved challenging, suggesting that a full-scale, NHS-based RCT would likely be unfeasible on the described design. Nonetheless, the trial provides critical insight into the design and implementation of future studies comparing these interventions.

Pleural infection remains a significant clinical concern across the NHS and worldwide. Unlike other respiratory infections, pleural infection outcomes have remained poor, with hospital length of stay averaging 13 days and mortality rates of 10–20%.^2^,^6^ Current standard management includes early intravenous antibiotics and chest drain insertion.

MT is a potentially advantageous alternative, supported by several European retrospective series. Ravaglia *et al* reported an 85.4% success rate among 41 patients with empyema managed with MT, rising to 91.7% in cases of multiloculated disease.^10^ Brutsche *et al* reported a 91% primary success rate in 127 patients across multiple centres,^13^ while Soler *et al* observed a 75% definitive cure rate in patients with complex disease, unresponsive to chest drains.^12^ Including seven additional patients reported by Colt,^11^ 172 of 191 patients (90%) across these series were successfully managed without surgery.

Despite its growing role in NHS practice, MT remains primarily a diagnostic tool for MPE,^7 9^ with variability in service provision arising from local needs and budgets. MT requires specialist staff, equipment and procedural space, all of which can delay timely intervention—a critical factor in pleural infection. While generally safe, MT necessitates sedation and is more time-consuming than chest drain insertion (43 vs 28 min in SPIRIT). Given this well-established alternative, physicians may be less willing to perform MT in very unstable patients. Coupled with a relative inability to evaluate these factors with a case series or audit, it further emphasised the need for a feasibility trial.

The primary outcome of trial feasibility was ultimately unsuccessful with only 6% of patients successfully prescreened. While 87 of 193 patients failed to meet standard diagnostic criteria for confirmed pleural infection, an additional 94 were excluded for reasons including the inability to deliver same-day or next-day MT, insufficient follow-up capacity and difficulties obtaining informed consent. These would all impede any larger trial. Although an alternative approach might have randomised all patients with suspected pleural infection, this would likely have led to high postrandomisation attrition rates due to the limited availability of urgent MT services in the UK. This would have raised ethical concerns regarding obtaining consent for an intervention known to be unavailable and would undermine an intention-to-treat analysis. While future studies could limit recruitment to centres with dedicated procedural suites, doing so here might have obscured a key barrier to wider implementation in subsequent research. Emergency lists may have facilitated rapid MT in endoscopy suites or theatres; however, these are generally reserved for acute pathology such as gastrointestinal bleeding or trauma and were felt to be impractical for the purposes of this study.

Even if all 193 patients had met eligibility criteria, 50 would have been excluded due to the centres’ inability to meet postprocedural requirements. This likely reflects limitations in necessary personnel (eg, ultrasound-trained staff) or logistical constraints for community-based follow-up. A more pragmatic, deprotocolised approach may reduce this prerandomisation attrition in future studies.

Of the 193 prescreened patients, we were unable to obtain informed consent in 34. Unfortunately, due to limited information provided at the time of prescreening, the precise reason was only available in two of 34 cases, with both being due to a lack of patient capacity. Although further patients would undoubtedly have also lacked capacity, it is highly likely that some proportion of the remaining 32 patients would have declined to receive the patient information sheet for unknown reasons or perhaps have declined participation in any research study offered to them. We have previously noted the latter occurring as a result of patients feeling overwhelmed by the stresses associated with concurrent illness and/or hospital admission. We would consider patients such as these distinct from those who would have been defined as screen failures in this study. Regardless, there remain challenges with undertaking interventional studies of all kinds in people who might be lacking capacity or who are frail and at greater risk of acute delirium. While alternative consent models (eg, waived, delayed or personal consultee consent) are gaining interest, their use is ethically constrained in trials involving invasive interventions when a safe, well-established alternative exists.

Importantly, despite failing to meet the composite feasibility outcome, there were no screen or allocation failures. This suggests that of those eligible and provided an information sheet, willingness to proceed to randomisation was generally high, and that this stage did not impose impactful delays on patient care. Furthermore, it supports that trial interventions were deliverable following randomisation. However, stringent eligibility criteria may have inadvertently excluded patients less likely to proceed to randomisation and to intervention, skewing this result.

While our trial was not feasible within the NHS framework at the time (2017–2019), a 2020 US study by Kheir *et al* assessed a similar approach.^15^ Patients with existing chest drains for pleural infection were received either MT or intrapleural fibrinolytic therapy (tPA and DNase). 48 hours between randomisation and procedure was allowed, giving greater logistical flexibility. Of 114 screened patients across three hospitals with immediate MT access, 32 (28%) were randomised. MT reduced postintervention hospital stay (median=2 vs 4 days; p=0.026) and improved microbiological yield,^16^ although it showed broadly similar treatment failure rates to intrapleural fibrinolytics (25% vs 19%).

While SPIRIT observed a longer total hospital stay in the MT vs the control arm (median=10 vs 8 days), both studies reported similar rates of treatment failure among MT patients (SPIRIT=33.3% vs US trial=25%). The higher recruitment rate in Kheir’s study (28% vs 6.2%) likely reflects the greater flexibility schedule and the use of MT as a second-line rather than first-line therapy. This might be more feasible within the UK by necessitating less urgent intervention, warranting prospective evaluation in an NHS setting. However, several recruitment barriers identified here (eg, contraindications to MT, lack of follow-up infrastructure and challenges with consent) would likely persist.

Additional data from Zhan *et al* in China further supports the role of MT in managing pleural infection.^17^ They retrospectively compared MT with intrapleural urokinase (n=33) versus chest drain with urokinase (n=75). MT involved thoracoscopic adhesiolysis and loculation breakdown prior to fibrinolytic administration. MT was associated with lower postoperative inflammatory markers, shorter antibiotic duration, reduced hospital stays and fewer initial treatment failures. This suggests MT could be a less invasive alternative to VATS for persistent infections, especially in frail patients unfit for a general anaesthetic. However, VATS remains superior in achieving complete debridement and has been associated with excellent outcomes.^18^ An ongoing RCT by Wang and colleagues may offer further insight when it concludes in December 2025.^19^

Brutsche *et al* reviewed 127 patients with multiloculated empyema treated with MT, approximately 1/3 of whom had failed prior drainage.^13^ As in SPIRIT, chest drains were left in place post MT until resolution. Further intervention was not required in 91% of participants, with a median drain duration of 7 days and median antibiotic duration of 10 days. These broadly align with our findings; in the intervention arm, five of six (83.3%) patients required no surgical intervention, median drain duration was 3 days and mean antibiotic duration was 7 days.

Fujita *et al* reported 30 Japanese patients undergoing MT for pleural infection, 60% (n=18) of whom were treated therapeutically.^20^ The success rate was high (94.4%), although hospital stay (median=23 days) and drain duration (median=9 days) were longer than observed here (median=10 days and 3 days, respectively). These differences may reflect cultural variations in inpatient care and a more severe disease focus of pyothorax in the Japanese cohort.

Although limited in scale and largely retrospective, the literature suggests MT is safe and technically feasible. While reinforced by SPIRIT, it suggests against a larger trial being feasible within the current NHS framework. A major barrier was an inability for most centres to deliver same- or next-day MT, largely reflecting the lack of dedicated pleural procedure suites for emergency intervention. This is particularly limiting given the urgency required in managing acute pleural infection. With one site accounting for 50% of enrolled patients, the trial would likely not be deliverable regardless of protocol design unless recruitment were to be restricted to centres with emergency MT capabilities. Similar challenges may be faced when looking to study specialist surgical procedures which can only be delivered in certain centres. For example, the recent MARS RCTs looked at the efficacy of surgical resection in mesothelioma and limited recruitment to only a handful of specific, expert locations.^21 22^ A similar approach may be theoretically feasible with acute MT; however, the urgency necessitated to deliver acute MT in the case of pleural infection would likely prevent recruitment-only centres from participating—as was the case with the MARS trials—therefore making achieving adequate power in such a study difficult. Additionally, findings in a limited context such as this would have poor generalisability across current NHS pleural service provision, necessitating substantial investment and systemic change. These challenges are intensified in the post-COVID-19 NHS landscape, where many centres have shifted towards day-case MT pathways to alleviate inpatient pressures. Expanding acute MT services would therefore place additional strain on already limited resources. In a redesigned trial, the inclusion criteria may be modified to allow for a more pragmatic approach to recruitment. With almost half of prescreened participants ineligible owing to a lack of hospital capacity for urgent MT, future studies may consider recruiting to MT intention and allowing for it within 72 hours of diagnosis, even with a drain in situ. Although this would shift the focus away from first-line drainage, it may be necessary to practically evaluate the therapeutic utility of MT in pleural infection in an NHS setting.

A key limitation was the considerable missing data among those who did not go on to receive study information (prescreen failures). Although recruitment sites were requested to gather details regarding this group, the level of data return was relatively poor. Gathering prestudy information can be difficult: in two previous RCTs led by our group, one-third of patients chose not to give a reason for turning down enrolment.^23 24^ In the SPIRIT trial, this effect was almost certainly magnified by needing to approach people who are acutely unwell, despite—paradoxically—this patient group potentially providing some of the most important information in the context of a feasibility study. Additionally, data completeness was poor among enrolled patients, particularly regarding follow-up thoracic ultrasound, with full adherence observed in only one of 12 participants. This likely reflects clinical pressures on study personnel and demonstrates the importance of feasibility trials in determining the processes of future studies.

Overall, SPIRIT was a multicentre feasibility study comparing MT to chest drain insertion for the initial management of pleural infection. While MT may be promising in selected contexts, a full-scale trial of this design is not currently feasible within the NHS. Future studies should consider our findings and pay particular attention to the wider clinical environment.

## Supplementary material

10.1136/bmjresp-2025-003675 online supplemental file 1

## Data Availability

Data are available upon reasonable request.
