# Supplementary material for: First-line medical thoracoscopy for pleural infection: the SPIRIT randomised controlled feasibility trial
Source: BMJ Open Respir Res. 2025 Dec 10;12(1):e003675. doi: 10.1136/bmjresp-2025-003675 (PMC12699672; doi:10.1136/bmjresp-2025-003675)
Supplement: online supplemental file 1 [file bmjresp-12-1-s001.pdf]

SUPPLEMENTARY DATA/INFORMATION

Supplement 1: Study recruitment flowchart

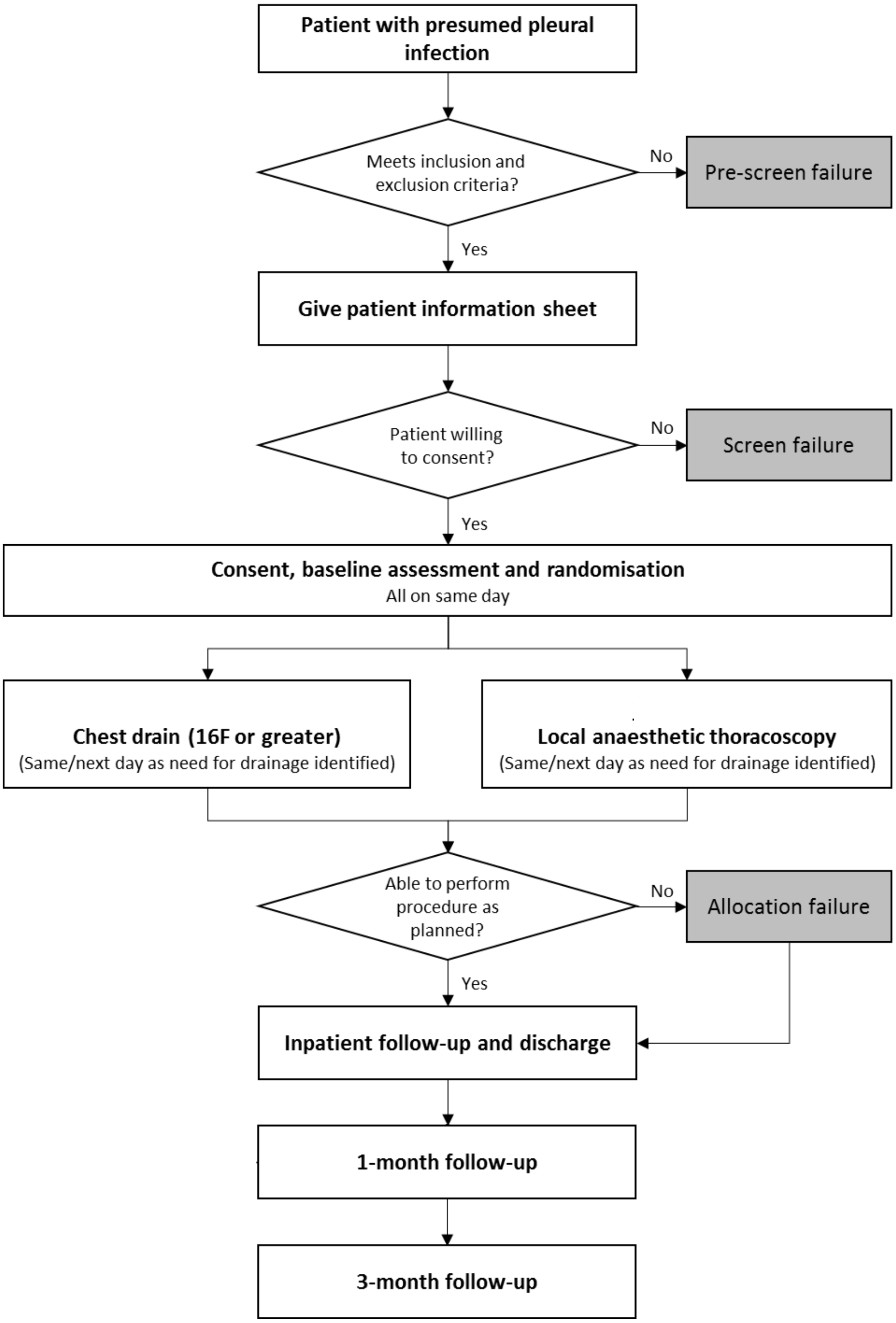

Supplement 2: Baseline demographic information

		All patients	Treatment group	
		Total N	Drain	LAT
			Count*	Count*
Admission to diagnosis delay, days (mean, SD)			2 (1)	1(1)
Admission to diagnosis delay, days (median, IQR))			2 (1,2)	0 (0,1)
Symptoms at diagnosis	No	1	0	1
	Yes	11	6	5
Duration of symptoms	Less than 1 week	1	0	1
	1-2 weeks	6	3	3
	2-4 weeks	1	1	0
	4-8 weeks	3	2	1
Smoking status	Never smoker	2	0	2
	Ex-smoker	4	3	1
	Current smoker	6	3	3
Alcohol intake	0 units per week	8	4	4
	1-10 units per week	1	0	1
	11-20 units per week	2	1	1
	>40 units per week	1	1	0
Previous IVDU	Yes	0	0	0
Received this year's infuenza vaccine	No	4	1	3
	Yes	6	4	2
	Unknown	2	1	1
Received pneumococcal vaccine	No	9	4	5
	Yes	1	1	0
	Unknown	2	1	1
Dental infection	Yes	0	0	0
Recent pneumonia	Yes	9	6	3
Recent sepsis	Yes	3	2	1
Recent chest trauma	Yes	0	0	0
Previous pleural infection (within last 6 weeks)	Yes	1	0	1
Recent diagnostic tap	Yes	0	0	0
Recent therapeutic tap	Yes	0	0	0
Recent dissection drain	Yes	0	0	0
Recent Seldinger drain	Yes	0	0	0
Recent pleural vent	Yes	0	0	0
Recent IPC insertion	Yes	0	0	0
Recent IPC drainage	Yes	0	0	0
Recent IPC removal	Yes	0	0	0
Recent thoracoscopy	Yes	0	0	0
Recent talc pleurodesis	Yes	0	0	0

Other recent procedure	Yes	0	0	0
Asthma	Yes	1	1	0
COPD	Yes	0	0	0
Interstitial lung disease	Yes	0	0	0
Bronchiectasis	Yes	0	0	0
Other respiratory history	Yes	1	1	0
Ischaemic heart disease	Yes	1	1	0
Heart failure	Yes	0	0	0
Atrial fibrillation	Yes	1	1	0
Other cardiac history	Yes	1	1	0
Recurrent UTIs	Yes	0	0	0
Chronic kidney disease	Yes	1	1	0
End-stage renal failure	Yes	0	0	0
Renal vasculitis	Yes	0	0	0
Other renal history	Yes	0	0	0
Viral hepatitis	Yes	0	0	0
Alcoholic hepatitis	Yes	1	1	0
Autoimmune hepatitis	Yes	0	0	0
Other hepatic history	Yes	1	0	1
Diabetes	Yes	4	4	0
Cerebrovascular disease	Yes	0	0	0
Active malignancy	Yes	0	0	0
Previous malignancy	Yes	1	1	0
Oral steroids	Yes	1	0	1
Inhaled steroids	Yes	1	1	0
Chemotherapy	Yes	0	0	0
Immunotherapy	Yes	1	0	1
Antiplatelets	Yes	0	0	0
Anticoagulants	Yes	0	0	0
Hb	124 (17)	111 (18)	117 (18)	
WBCs	17.6 (11.7)	11.9 (6.4)	14.7(9.4)	
Neutrophils	15.1 (11.9)	9.5 (5.6)	12.3 (9.3)	
Lymphocytes	1.34 (0.48)	1.18 (0.65)	1.26 (0.55)	
Platelets	322 (185)	466 (173)	394 (185)	
Urea	6.54 (4.07)	4.42 (1.48)	5.6 (3.2)	
Albumin	31.6 (7.7)	36.5 (2.1)	33.6 (6.2)	

*Unless stated

	Control arm (n=6)	Intervention arm (n=6)	All (n=12)
Positive fluid culture	1	2	3
Positive pleural tissue culture	N/A	1	1

Supplement 3:

6 of the 12 participants (50%) displayed complete adherence with the study protocol and had chest radiographs the 5 specified time-points. 7 of the 12 participants (58.3%) had chest radiographs performed post-procedure. Supplement 3, Table 1 displays the complete data on when participants had follow-up radiographic chest imaging as per the trial schedule.

Supplement 3, Table 1: Adherence to chest radiograph schedule

	Participants with chest radiographs at each time point = N (%)
Baseline	11 (91.7)
Post-procedure	7 (58.3)
Discharge	10 (83.3)
Day 30	9 (75)
Day 90	8 (66.7)

1 of the 12 participants (8.3%) had complete adherence with the study protocol and had thoracic ultrasound performed at the 6 specified time points. 2 of the 12 participants had no repeat ultrasound imaging following baseline assessment throughout the 90-day follow-up period. Supplement 3, Table 2 displays the complete data on when participants had follow-up thoracic ultrasound imaging as per the trial schedule.

Supplement 3, Table 2: Adherence to thoracic ultrasound imaging schedule

	Participants with thoracic ultrasounds performed at each time point = N (%)
Baseline	12 (100)
Day 1	5 (41.6)

Day 3	6 (50)
Day 7	8 (66.7)
Day 30	8 (66.7)
Day 90	7 (58.3)

Supplement 4:

Patient reported EQ-5D and VAS at baseline and follow-up.

		**Baseline**	**7 days**	**30 days**	**90 days**
**Control arm**	EQ5D, median (IQR)	0.65 (0.58)	0.74 (0.29)	0.80 (0.27)	0.74 (0.24)
	Chest pain (mm)	40.17 (23.6)	12.50 (12.0)	10.67 (10.0)	n/a
	Breathlessness (mm)	49.67 (30.7)	16.33 (16.2)	18.67 (16.9)	n/a
**Intervention arm**	EQ5D, median (IQR)	0.50 (0.63)	0.49 (0.39)	0.68 (0.22)	0.77 (0.48)
	Chest pain (mm)	18.50 (26.4)	3.75 (2.2)	12.60 (10.8)	n/a
	Breathlessness (mm)	40.67 (41.7)	17.75 (19.7)	15.80 (19.7)	n/a

All figures recorded represent the mean (SD), unless otherwise specified.

Supplement 5:

Adverse event data.

	**All patients (n-12)**	**Control arm (n-6)**	**Intervention arm (n-6)**
Total AEs	12	1	11*
Number of patients with AE	4	1	3
Mild category	9	0	9
Moderate category	2	0	2
Severe category**	1	1	0
Pain due to intervention	2	0	2
Drain blockage	1	0	1
Surgical emphysema	1	0	1
Intervention (dislodgement)	2	0	2
Tachycardia	1	0	1
Sepsis	1	0	1
Fever	1	0	1
Death due to comorbidity**	1	1	0
Other surgical complication	2	0	2
Total SAEs	1	1	0

*8 of these events occurred in one patient
**Patient death due to underlying malignancy; reported as SAE also
